# A method of predicting the best conditions for large-size workpiece clamping to reduce vibration in the face milling process

**DOI:** 10.1038/s41598-021-00128-6

**Published:** 2021-10-21

**Authors:** Krzysztof J. Kaliński, Natalia Stawicka-Morawska, Marek A. Galewski, Michał R. Mazur

**Affiliations:** Faculty of Mechanical Engineering and Ship Technology, Institute of Mechanics and Machine Design, Gdansk University of Technology, 80-233 Gdansk, Poland

**Keywords:** Engineering, Mechanical engineering

## Abstract

The paper presents an innovative method of solving the problem of vibration suppression during milling of large-size details. It consists in searching for the best conditions for clamping the workpiece based on a rapid modal identification of the dominant natural frequencies only and requires repetitive changes in the tightening torque of the clamping screws. Then, by estimating the minimum work of the cutting forces acting in the direction of the width of the cutting layer, it is possible to predict the best fixing of the workpiece. Application of the method does not require the creation and identification of a computational model of the process or preliminary numerical simulations. The effectiveness of this method was confirmed by the evaluation of the Root Mean Square (RMS) of the vibration level in the time domain observed during the actual face milling process. The worst results were obtained for the configuration of supports tightened with a torque of 90–110 Nm, and the best—with a torque of 50 Nm.

## Introduction

The main reason of various problems in the machining process of large-size structures are the relative vibrations of the tool-workpiece^1^. Their presence results, in order to prevent deterioration of the quality of the machined surface, in the necessity to limit the overall productivity of machine tools^2–4^. Moreover, increased wear and, in extreme cases, destruction of the tool or workpiece are observed^5^. In order to reduce the level of vibrations and thus - to ensure the required surface quality, it was proposed a method of adjusting the rotational speed of the tool to the optimal angle of phase shift between the inner and outer modulation of the thickness of the cutting layer^6^, which speed also results from the condition of minimizing the work of cutting forces in the direction of the layer thickness^7,8^.

Machine tools developed specifically for large workpieces are characterized by the fact that the operation of general engineering principles differs significantly from those used on conventional size machines. The demand for the production of large parts is generally increasing, while the current scientific research results lag behind and are usually far from the expected requirements in this field^9^. Numerous methods make it possible to search for the optimal level of vibrations by considering mainly the phenomena observed in the direction of the thickness of the cutting layer^2–4,7,10,11^. The latter also applies to the issues of milling large-size objects, important from the point of view of milling force prediction, machining dynamics including vibration suppression, as well as part error and surface quality^1^. Machining deformation of structural parts was a serious issue in part quality control, with particular emphasis on the machining sequence adjustment to control deformation by taking advantage of a responsive fixture based machining method^12^. Deformation data can be monitored during the machining process, so that the in-process state of the workpiece can be obtained online. Despite the promising results, in the future, attention should be paid to adjusting the machining sequence to change the machining depth in each individual layer, and adjusting the machining sequence for different kinds of part structures and more complex constraints. Predicting the dynamics of a flexible workpiece is a critical factor in milling large-scale thin-walled structures, and an efficient decomposition-condensation method was developed for this purpose^13^. The conducted material experiments have shown that the proposed method, together with the dynamic model, can accurately predict the stability of the milling process of structures with planes or curved surfaces. In the pocket milling process, the most flexible point on the workpiece is the center wall area, and this is where the stability should be carefully checked when planning the process. The above approaches are useful when chatter is predominant.

Due to the more complicated nature of vibrations during the large-size milling process, the recommended solution cannot be associated only with the chatter vibration phenomenon. Therefore, a dedicated approach should take into account not only the natural frequencies accompanying the identified poles of the system, but also the more important and more intense influence of harmonic frequencies of forced vibrations^14^. Nevertheless, the importance of forced vibrations in the machining of large-size workpieces has already been noticed^9^, and due to the variable thickness of chips and the discontinuous nature of the process, they always occur. While the improvements in recent years have been amazing, there are still many challenges left, and plenty of room for further research. Of all possible research topics, it can be concluded that those concerning the precision of machining and the reduction of cycle time may have the highest priority^9^.

The subject of the paper is a method of searching for the conditions for minimizing the vibration level of a tool-large size flexible workpiece, with the same technological parameters of the milling process, but with different workpiece clamping conditions, resulting in different stiffness. Thus, it is a special case of the formulation of a problem specific to semi-active systems, although the control signals are not explicitly present here. The problem of ensuring the correct stiffness of the machine tool foundation for machining large-size details has been solved for example by the optimized sequence of tightening the anchor bolts for a given configuration of the machine bed and anchor system^15^. However, in order to improve the surface quality during the face milling process, it is more important to optimize the mounting pattern of the workpiece on the machine table based on the structure of variable stiffness^16^. Also, various workpiece holders can affect energy consumption, tool wear, and surface quality in milling operations^17^. For example, increasing the stiffness of the spindle system in the direction of the feed rate can effectively improve the stability in the up-milling process, while the opposite is true in the down-milling process^18^. Stiffness changes for the two degrees of freedom of a rather smaller milling system are also performed by piezoelectric stack actuators acting on a rotating tool^19^.

The developed method presented in this paper is different from the ones described in the above-cited literature because it is assumed that the dominant direction of vibrations is the width of the cutting layer, which has a significant impact on the quality of the product. Minimizing the work of cutting forces in the direction of the layer width corresponds to the best conditions for clamping the workpiece, because in the case of stable machining (no self-excited vibrations), minimizing the level of tool-workpiece vibrations in this direction is of key importance for obtaining the required accuracy and quality (roughness) of the machined surface. This was confirmed by the results of experimental tests of the face milling process presented in the article. The original works cited earlier^7,8^ concern the minimization of the cutting forces, but in the direction of the layer thickness. This prevents the loss of stability and the formation of self-excited chatter vibrations. The considerations of this article concern stable machining; chatter vibrations do not occur at all here. Hence, the subject of works^6–8^ is not applicable in the current considerations. It is much more important to minimize vibrations towards the width of the layer. The authors are not familiar with the previous literature studies on the optimization of cutting conditions related to the work of cutting forces in the direction of the layer width.

## Methods

The proposed method consists in determining the best conditions for clamping the workpiece with the use of rapid modal analysis of the workpiece, performed only to identify its dominant natural frequencies. However, the effective application of the developed solution requires the provision of repeatable conditions for fixing the object, e.g. by measuring the tightening torque of the mounting screws with a dynamometric spanner. The proposed method of determining the influence of the tightening torque consists in linking its value with the natural frequencies of the object mounted on the machine table, identified on the basis of the experimentally determined Frequency Response Function (FRF). It is a credible assessment because experimental tests have shown that the condition of fastening repeatability is met, especially when determining the frequency of the maximums of the FRF characteristic. In this way, it is possible to experimentally determine, for a given workpiece, a family of static characteristics "tightening torque - natural frequencies", showing the influence of the tightening torque on the dynamics of the tested system. The latter also makes it attractive from an economic point of view. Due to the fact that knowledge of the computational model of the machining process is not required, the implementation of the solution can take place with minimal financial outlays. The prior approach of completely loosening and re-tightening the next screws^20^ is of less practical importance due to the risk of failure to repeat the fastening state.

**Figure 1:**
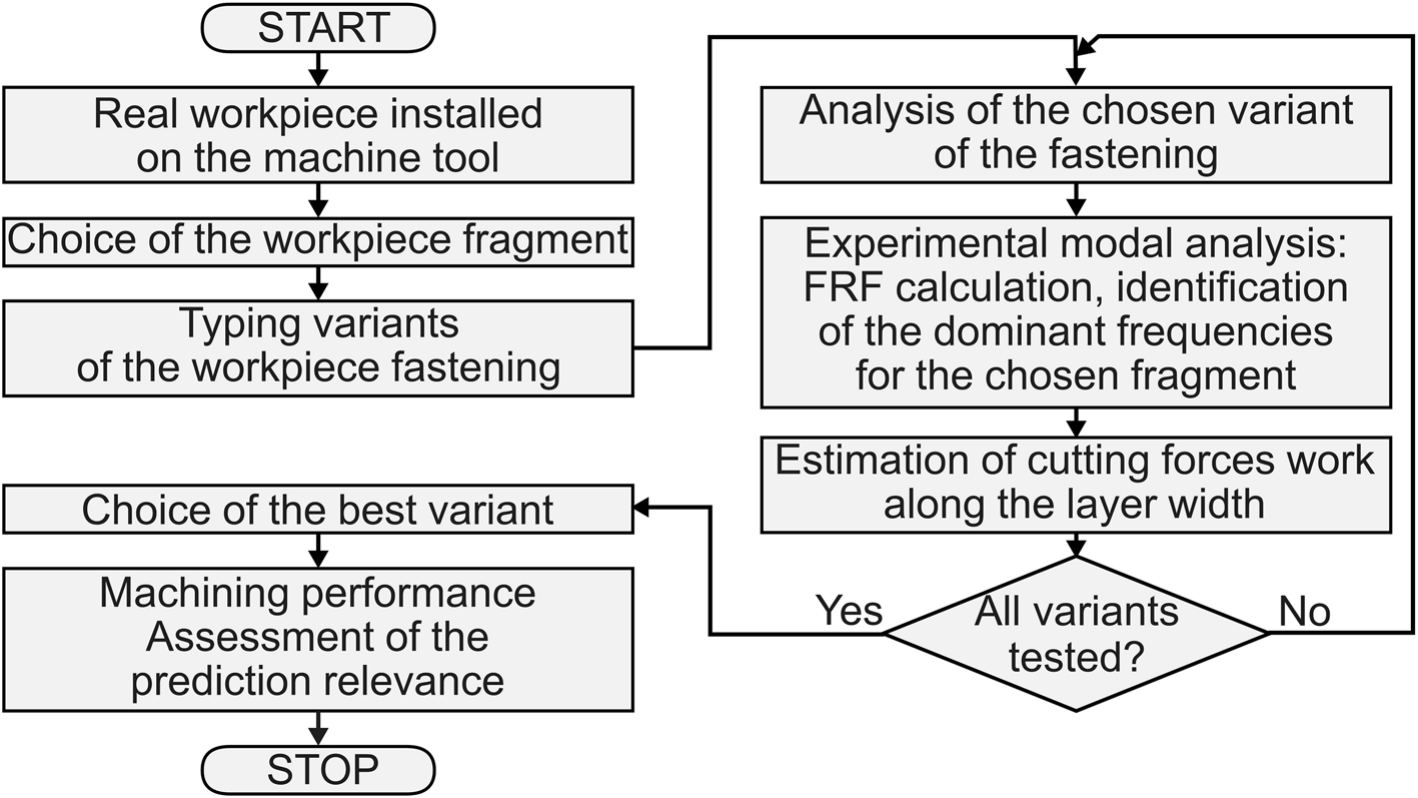
Scheme for finding the best conditions for fixing the workpiece by minimizing the work of cutting forces.

The developed innovative solution is based on (Fig. 1):

- Measurement of impulse vibration characteristics (FRFs) in a previously selected part of the workpiece mounted on a machine tool and determination of the frequency of the dominant peaks in the amplitude spectrum;
- Determination of the best variant of clamping the workpiece from the condition of estimating the minimum work of cutting forces in the direction of the width of the cutting layer. This is an original, so far unpublished proprietary approach. It is an extension of the idea presented in the patent description^21^, due to taking into account the work of cutting forces coming from a finite number of teeth of the tool currently in contact with the workpiece;
- Implementation of the machining process according to the best variant of the workpiece clamping.

The motivation to calculate the work of the cutting force in the direction of the width of the layer (correlated with the depth of cutting) is due to the fact that this direction is consistent with the direction of normal vibrations to the machined surface, the level of which directly determines the surface quality (geometric accuracy, roughness). The lower the vibration level in the direction of the layer width, the better the quality of the machined surface is observed. This was confirmed by the results of experimental tests (vibration measurements) during the considered milling processes.

### Cutting process dynamics

**Figure 2:**
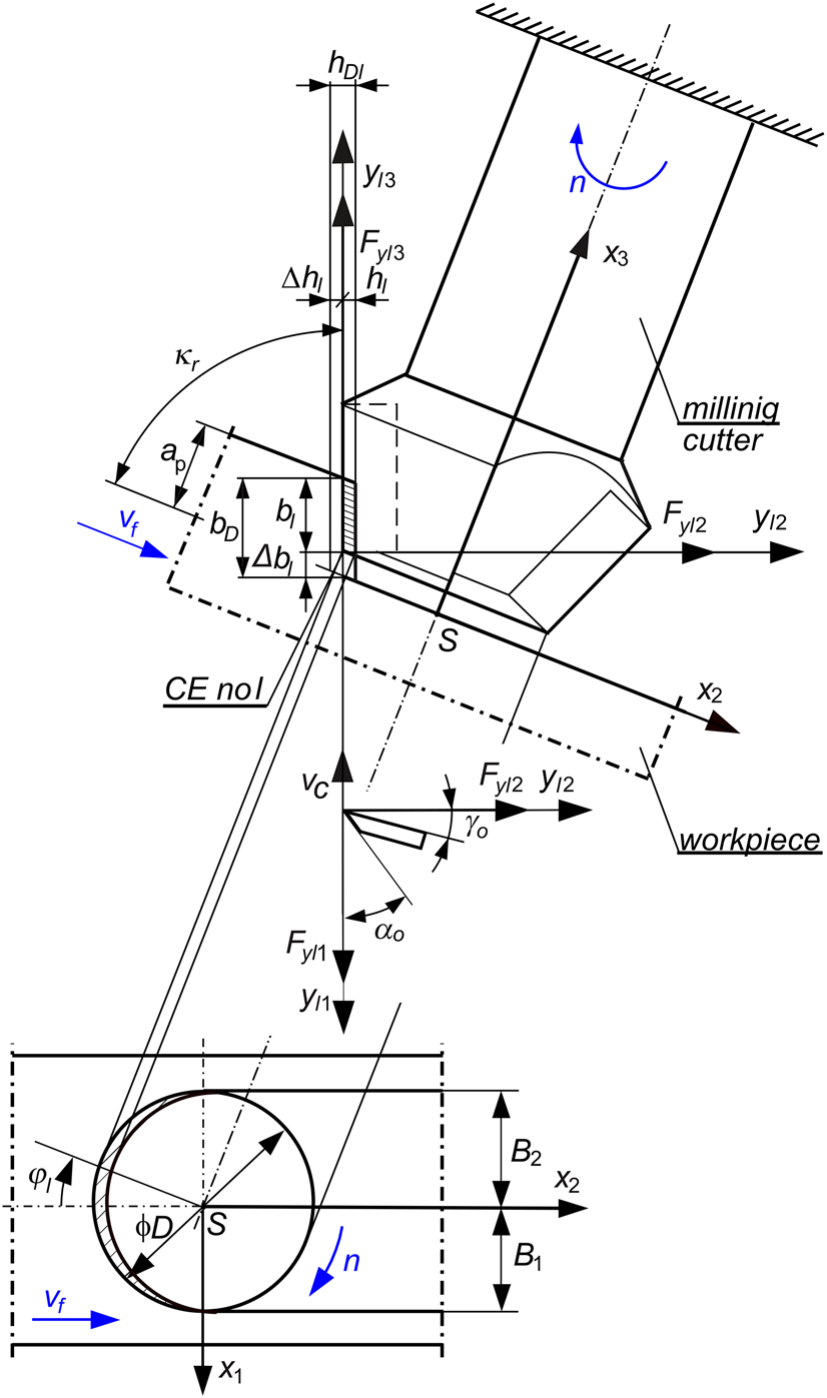
Scheme of a face milling of a large-size flexible workpiece.

A dynamic analysis of the face milling process of a large-size flexible workpiece (Fig. 2), was carried out based on the following assumptions^8,22^.

- The spindle, together with the milling cutter (tool) fixed in the holder, and the table with the workpiece, are separated from the machine tool structure. The constant spindle speed is *n*, while the feed speed of the workpiece is *v*_*f*_. The remaining parts of the milling machine are recognized as ones whose influence can be neglected^7,9,10^.
- Only the flexibility of the workpiece is considered. The latter applies in particular to a large-size flexible workpiece processed with a rigid tool^1,13^.
- Coupling Elements (CEs) are used to model the dynamic interaction of the cutting process between the edges of the selected teeth and the workpiece.
- An effect of first pass of the tooth’s edge along cutting layer causes proportional feedback, and the effect of multiple passes causes delayed feedback additionally.

Mechanistic modeling of the cutting force is applied to estimate the components of the cutting force ^1,23^. Thus, for instantaneous contact point between the chosen tool edge and the workpiece (idealized by CE no. *l*), proportional model of the cutting dynamics is included^8,22^. In contrast with the previous considerations^7,24^, the three-dimensional proportional model of the machining process dynamics was adopted^14^. Based on this model, the cutting forces depend proportionally on instantaneous cutting layer thickness *h*_*l*_(*t*), and also on instantaneous cutting layer width *b*_*l*_(*t*); both of them vary in time. According to the direction of the action, we separate cutting force component *F*_*yl*1_ acting along nominal cutting speed *v*_*c*_, cutting force component *F*_*yl*2_ acting along cutting layer thickness, and additionally—cutting force component *F*_*yl*3_ acting along cutting layer width. These cutting force components are described by relationships^14,25^:

1 $$F_{yl1} \left( t \right) = \left\{ {\begin{array}{*{20}l} {k_{dl} b_{l} \left( t \right)h_{l} \left( t \right),} & {h_{l} \left( t \right) > 0 \wedge b_{l} \left( t \right) > 0,} \\ {0,} & {h_{l} \left( t \right) \le 0 \vee b_{l} \left( t \right) \le 0,} \\ \end{array} } \right.$$

2 $$F_{yl2} \left( t \right) = \left\{ {\begin{array}{*{20}l} {\mu_{l2} k_{dl} b_{l} \left( t \right)h_{l} \left( t \right),} & {h_{l} \left( t \right) > 0 \wedge b_{l} \left( t \right) > 0,} \\ {0,} & {h_{l} \left( t \right) \le 0 \vee b_{l} \left( t \right) \le 0,} \\ \end{array} } \right.$$

3 $$F_{yl3} \left( t \right) = \left\{ {\begin{array}{*{20}l} {\mu_{l3} k_{dl} b_{l} \left( t \right)h_{l} \left( t \right),} & {h_{l} \left( t \right) > 0 \wedge b_{l} \left( t \right) > 0,} \\ {0,} & {h_{l} \left( t \right) \le 0 \vee b_{l} \left( t \right) \le 0,} \\ \end{array} } \right.$$

where:

4 $${b}_{l}\left(t\right)={b}_{D}-\Delta {b}_{l}\left(t\right),$$

5 $${h}_{l}\left(t\right)={h}_{Dl}\left(t\right)-\Delta {h}_{l}\left(t\right)+\Delta {h}_{l}\left(t-{\tau }_{l}\right),$$

*b*_*D*_—desired cutting layer width, *b*_*D*_ = *a*_*p*_/sin*κ*_*r*_, Δ*b*_*l*_(*t*)—dynamic change in cutting layer width for CE no. *l*, *h*_*Dl*_(*t*)—desired cutting layer thickness for CE no. *l*, *h*_*Dl*_(*t*) ≅ *f*_*z*_ sin*κ*_*r*_ cos*φ*_*l*_(*t*), Δ*h*_*l*_(*.*)—dynamic change in cutting layer thickness for CE no. *l*, *k*_*dl*_—average dynamic specific cutting pressure for CE no. *l*, *μ*_*l*2*,*_* μ*_*l*3_—cutting force ratios for CE no. *l*, as quotients of forces *F*_*yl*2_ and *F*_*yl*1_, and forces *F*_*yl*3_ and *F*_*yl*1_, *τ*_*l*_—time-delay between the same position of CE no. *l* and of CE no. *l*-1, *a*_*p*_—desired depth of cutting, *κ*_*r*_—cutting edge angle, *γ*_o_—rake angle in orthogonal plane of the edge, *α*_o_—clearance angle in orthogonal plane of the edge, *φ*_*l*_(*t*)—immersion angle of tooth no. *l*, i.e. angular position of CE no. *l*, *f*_*z*_—feed per tooth; *f*_*z*_ = *v*_*f*_ /(*zn*)*, z*—number of teeth of the milling tool.

The description of cutting forces for CE no. *l* in six-dimensional space takes the form^14^:

6 $${\mathbf{F}}_{l}\left(t\right)={\mathbf{F}}_{l}^{0}\left(t\right)-{\mathbf{D}}_{Pl}\left(t\right)\Delta {\mathbf{w}}_{l}\left(t\right)+{\mathbf{D}}_{Ol}\left(t\right)\Delta {\mathbf{w}}_{l}\left(t-{\tau }_{l}\right),$$

where:

7 $${\mathbf{F}}_{l}\left(t\right)=col({F}_{yl1}\left(t\right),{F}_{yl2}\left(t\right),{F}_{yl3}\left(t\right),\mathrm{ 0,0},0),$$

8 $${\mathbf{F}}_{l}^{0}\left(t\right)=col({k}_{dl}{b}_{D}{h}_{Dl}\left(t\right),{{\mu }_{l2}k}_{dl}{b}_{D}{h}_{Dl}\left(t\right),{{\mu }_{l3}k}_{dl}{b}_{D}{h}_{Dl}\left(t\right),\mathrm{ 0,0},0),$$

9 $${\mathbf{D}}_{Pl}\left(t\right)=\left[\begin{array}{ll}\begin{array}{ccc}0& {k}_{dl}\left({b}_{D}-\Delta {b}_{l}\left(t\right)\right)& {k}_{dl}{h}_{Dl}\left(t\right)\\ 0& {{\mu }_{l2}k}_{dl}\left({b}_{D}-\Delta {b}_{l}\left(t\right)\right)& {{\mu }_{l2}k}_{dl}{h}_{Dl}\left(t\right)\\ 0& {{\mu }_{l3}k}_{dl}\left({b}_{D}-\Delta {b}_{l}\left(t\right)\right)& {{\mu }_{l3}k}_{dl}{h}_{Dl}\left(t\right)\end{array}& {\mathbf{0}}_{3\times 3}\\ {\mathbf{0}}_{3\times 3}& {\mathbf{0}}_{3\times 3}\end{array}\right],$$

10 $${\mathbf{D}}_{Ol}\left(t\right)=\left[\begin{array}{ll}\begin{array}{ccc}0& {k}_{dl}\left({b}_{D}-\Delta {b}_{l}\left(t\right)\right)& 0\\ 0& {{\mu }_{l2}k}_{dl}\left({b}_{D}-\Delta {b}_{l}\left(t\right)\right)& 0\\ 0& {{\mu }_{l3}k}_{dl}\left({b}_{D}-\Delta {b}_{l}\left(t\right)\right)& 0\end{array}& {\mathbf{0}}_{3\times 3}\\ {\mathbf{0}}_{3\times 3}& {\mathbf{0}}_{3\times 3}\end{array}\right],$$

11 $${\Delta \mathbf{w}}_{l}\left(t\right)=col({q}_{zl}\left(t\right),\Delta {h}_{l}\left(t\right),\Delta {b}_{l}\left(t\right),\mathrm{0,0},0),$$

12 $${\Delta \mathbf{w}}_{l}\left(t-{\tau }_{l}\right)=col\left({q}_{zl}\left(t-{\tau }_{l}\right),\Delta {h}_{l}\left(t-{\tau }_{l}\right),\Delta {b}_{l}\left(t-{\tau }_{l}\right),\mathrm{0,0},0\right),$$

and: ${q}_{zl}\left(t\right)$—relative displacement of edge tip and workpiece along direction *y*_*l*1_ at instant of time *t*, ${q}_{zl}\left(t-{\tau }_{l}\right)$– relative displacement of edge tip and workpiece along direction *y*_*l*1_ at instant of time *t*—*τ*_*l*_.

### Minimizing the work of cutting forces

A method is being sought to minimize the level of tool–workpiece vibrations. The only source of energy supporting the vibration is the work of forces in the cutting process. Hence, one should strive to meet such process conditions that the work is minimal. If the number of *i*_*l*_ teeth of the cutting tool is taken into account, we determine this work from the relationship^7,8^:

13 $$L\left(t\right)=\sum_{l=1}^{{i}_{l}}\int {\mathbf{F}}_{l}^{T}\left(t\right)d{\Delta \mathbf{w}}_{l},$$

and then, after considering the expression (6):

14 $$L\left(t\right)=\sum_{l=1}^{{i}_{l}}\int \left({\left({\mathbf{F}}_{l}^{0}\left(t\right)\right)}^{T}-{\Delta \mathbf{w}}_{l}^{T}{\left(t\right)\mathbf{D}}_{Pl}^{T}\left(t\right)+{\Delta \mathbf{w}}_{l}^{T}{\left(t-{\tau }_{l}\right)\mathbf{D}}_{Ol}^{T}\left(t\right)\right)d{\Delta \mathbf{w}}_{l}.$$

The above equation is universal, because it takes into account the work of cutting forces in different directions, as well as the non-stationarity and non-linearity of the computational model. The latter considerably complicates the problem of searching for an unambiguous condition for minimizing the vibration level. To deal with this, it is proposed to make some simplifications, namely:

- Consideration of the temporary stationary positions of the teeth of a cutting tool with a uniform pitch, relative to the workpiece in the extreme unfavorable case. Of course, this is a certain simplification of the time-varying milling process, because we intend to operate a description that ignores the change of the structure configuration over time;
- Assuming the hypothesis that the level of vibration of the workpiece in the direction normal to the machined surface is determined by the work of the cutting force of edge no. *l* in the direction of the width of the layer *y*_*l*3_, and the work in the other directions, i.e. thickness of the layer *y*_*l*2_ and cutting speed *y*_*l*1_, is omitted;
- The influence of dynamic changes in layer thickness (see, Eq. (5)) is ignored, i.e. ${h}_{l}\left(t\right)={h}_{Dl}\left(t\right)$. Observations made during the measurement of vibrations of the tested cases of milling flat surfaces showed that in each of them we are dealing with stable machining; chatter vibrations do not occur at all. The above justifies the desirability of ignoring the regenerative vibrations in the direction of the layer thickness, in particular the regeneration effect of the trace, which is a potential cause of loss of stability and the occurrence of self-excited chatter vibrations;
- In the case of machining large-size workpieces, the vibration level in the direction of the layer width is much lower than its nominal value, i.e. $\left|{\Delta b}_{l}\left(t\right)\right|\ll {b}_{D}$;
- From the mathematical point of view, the energy dissipation effect was omitted in the considerations.

Based on the above assumptions, the instantaneous layer thickness depends only on the kinematic cutting conditions, i.e. it is a function of the frequency of the cutting edge entering the material:

15 $${h}_{Dl}\left(t\right)={f}_{z}\mathrm{sin}{\upkappa }_{r}\mathrm{cos}\left(\frac{2\pi n}{60}t+{\varphi }_{1}+\left(l-1\right)\frac{2\pi }{z}\right),$$

where: ${\varphi }_{1}$—immersion angle of tooth no. 1 (i.e. angular position of CE no. 1), being in contact with the workpiece, and the expressions (11) and (12) will take the forms respectively:

16 $${\Delta \mathbf{w}}_{l}\left(t\right)=col({q}_{zl}\left(t\right),0,\Delta {b}_{l}\left(t\right),\mathrm{0,0},0),$$

17 $${\Delta \mathbf{w}}_{l}\left(t-{\tau }_{l}\right)=col({q}_{zl}\left(t-{\tau }_{l}\right),0,\Delta {b}_{l}\left(t-{\tau }_{l}\right),\mathrm{0,0},0).$$

Then we calculate the differential of expression (16), that is to say:

18 $$d{\Delta \mathbf{w}}_{l}\left(t\right)=col(0, 0,d\Delta {b}_{l}\left(t\right),\mathrm{0,0},0).$$

After taking into account equations (7), (8) and (14), (15), (16), (17) and (18), the work of forces after taking into account *i*_*l*_ cutting edges in the cutting process will take the form:

19 $$\begin{aligned} L\left( t \right) & = \sum\limits_{l = 1}^{{i_{l} }} {\int {\mu_{3l} k_{dl} f_{z} \sin \kappa_{r} \cos \left( {\frac{2\pi n}{{60}}t + \varphi_{1} + \left( {l - 1} \right)\frac{2\pi }{z}} \right)\left( {b_{D} - \Delta b_{l} \left( t \right)} \right)d\Delta b_{l} \left( t \right)} } \mathop \Rightarrow \limits^{{\left| {\Delta b_{l} \left( t \right)} \right| \ll b_{D} }} \\ & \cong \sum\limits_{l = 1}^{{i_{l} }} {\int {\mu_{3l} k_{dl} f_{z} \sin \kappa_{r} b_{D} \cos \left( {\frac{2\pi n}{{60}}t + \varphi_{1} + \left( {l - 1} \right)\frac{2\pi }{z}} \right)d\Delta b_{l} (t)} } . \\ \end{aligned}$$

We observe here a complex state of vibrations excited by variable forces, depending on changes in the direction of the thickness of the layer with the frequency of the cutting edge entering the material, and in the direction of the layer width—with a combination of natural vibrations of the tool-workpiece, i.e.:

20 $${\Delta b}_{l}\left(t\right)=\sum_{\alpha =1}^{{i}_{\alpha }}{\Delta b}_{l\alpha }\left(t\right),$$

where:

21 $${{\Delta b}_{l\alpha }\left(t\right)=\Delta b}_{l\alpha }^{0}\mathrm{sin}\left({\omega }_{\alpha }t\right),$$

${\Delta b}_{l\alpha }^{0}$, ${\omega }_{\alpha }$—amplitude and angular frequency of natural vibration component no. $\alpha$, observed for tooth (CE) no. *l*.

Calculating the differential of expression (20), we get:

22 $${d\Delta b}_{l}\left(t\right)=\sum_{\alpha =1}^{{i}_{\alpha }}{d\Delta b}_{l\alpha }\left(t\right)=\sum_{\alpha =1}^{{i}_{\alpha }}{\Delta b}_{l\alpha }^{0}{\omega }_{\alpha }\mathrm{cos}\left({\omega }_{\alpha }t\right)dt,$$

and subsequently—work of cutting forces:

23 $$\begin{aligned} L\left( t \right) & = \sum\limits_{l = 1}^{{i_{l} }} {\int {\mu_{3l} k_{dl} f_{z} \sin_{r} b_{D} \cos \left( {\frac{2\pi n}{{60}}t + \varphi_{1} + \left( {l - 1} \right)\frac{2\pi }{z}} \right)\sum\limits_{\alpha = 1}^{{i_{\alpha } }} {\Delta b_{l\alpha }^{0} \omega_{\alpha } \cos \left( {\omega_{\alpha } t} \right)dt} } } \\ & = \sum\limits_{\alpha = 1}^{{i_{\alpha } }} {\sum\limits_{l = 1}^{{i_{l} }} {\int{\mu_{3l} k_{dl} f_{z} \sin \kappa_{r} b_{D} \Delta b_{l\alpha }^{0} \omega_{\alpha } \cos \left( {\frac{2\pi n}{{60}}t + \varphi_{1} + \left( {l - 1} \right)\frac{2\pi }{z}} \right)\cos (\omega_{\alpha } t)dt} } }. \\ \end{aligned}$$

Due to the complex nature of the exciting force, it is impossible to separate the steady-state forced vibrations and transient vibrations in the cutting process. The above means that the assigned work (23) has a periodic character. Thus, if the optimality conditions are determined for an assumed undamped system, they will also be met when energy dissipation occurs in the system.

Based on Eq. (23) we calculate the work of the cutting forces of ${i}_{l}$ teeth being in contact with the workpiece during one period of only harmonic vibrations with angular frequency ${\omega }_{\alpha }$:

24 $$\begin{aligned} L_{\alpha } & = \sum\limits_{l = 1}^{{i_{l} }} {\int\limits_{0}^{{\frac{2\pi }{{\omega_{\alpha } }}}} {\mu_{3l} k_{dl} f_{z} \sin \kappa_{r} b_{D} \Delta b_{l\alpha }^{0} \omega_{\alpha } \cos \left( {\frac{2\pi n}{{60}}t + \varphi_{1} + \left( {l - 1} \right)\frac{2\pi }{z}} \right)} \cos \left( {\omega_{\alpha } t} \right)dt} \\ & = \sum\limits_{l = 1}^{{i_{l} }} {\mu_{3l} k_{dl} f_{z} \sin \kappa_{r} b_{Dl} \Delta b_{l\alpha }^{0} \omega_{\alpha } \int\limits_{0}^{{\frac{2\pi }{{\omega_{\alpha } }}}} {\cos \left( {\frac{2\pi n}{{60}}t + \varphi_{1} + \left( {l - 1} \right)\frac{2\pi }{z}} \right)\cos \left( {\omega_{\alpha } t} \right)dt.} } \\ \end{aligned}$$

After transformation, the definite integral in formula (24) will take the form:

25 $$\underset{0}{\overset{\frac{2\pi }{{\omega }_{\alpha }}}{\int }}\mathrm{cos}\left(\frac{2\pi n}{60}t+{\varphi }_{1}+\left(l-1\right)\frac{2\pi }{z}\right)\mathrm{cos}({\omega }_{\alpha }t)dt=\frac{1}{2}\frac{\frac{4\pi n}{60}}{{\left(\frac{2\pi n}{60}\right)}^{2}-{\omega }_{\alpha }^{2}}\left[\mathrm{sin}\left(\frac{4{\pi }^{2}n}{60{\omega }_{\alpha }}+{\varphi }_{1}+\left(l-1\right)\frac{2\pi }{z}\right)-\mathrm{sin}\left({\varphi }_{1}+\left(l-1\right)\frac{2\pi }{z}\right)\right].$$

Hence, after taking the dependence (25) into account, the work of the cutting forces (24) will be:

26 $${L}_{\alpha }=\sum_{l=1}^{{i}_{l}}\left[{\upmu }_{3l}{k}_{dl}{f}_{z}{\mathrm{sin}{\upkappa }_{r}b}_{D}{\Delta b}_{l\alpha }^{0}{\omega }_{\alpha }\cdot \frac{\frac{\pi n}{30}}{{\left(\frac{2\pi n}{60}\right)}^{2}-{\omega }_{\alpha }^{2}}\cdot \left(\mathrm{sin}\left(\frac{4{\pi }^{2}n}{60{\omega }_{\alpha }}+{\varphi }_{1}+\left(l-1\right)\frac{2\pi }{z}\right)-\mathrm{sin}\left({\varphi }_{1}+\left(l-1\right)\frac{2\pi }{z}\right)\right)\right].$$

If we assume the duration of the process *T*, then the work of component no. *α* performed during this time will be:

27 $${\widehat{L}}_{\alpha }=\frac{T}{\frac{2\pi }{{\omega }_{\alpha }}}{L}_{\alpha }=\frac{T{\omega }_{\alpha }}{2\pi }{L}_{\alpha }.$$

The duration of the process *T* should be relatively short so that the assumed instantaneous positions of the tool edges do not change significantly.

On the other hand, the work performed by all the components ${i}_{\alpha }$ during the duration of process *T* will take the form:

28 $$\begin{aligned} L &= \sum\limits_{ \propto = 1}^{{i_{ \propto } }} {\hat{L}_{ \propto } = \frac{T}{2\pi }} \sum\limits_{ \propto = 1}^{{i_{ \propto } }} {L}_{ \propto } \omega_{\alpha } = \frac{{Tf_{z} }}{2\pi }\sum\limits_{ \propto = 1}^{{i_{ \propto } }} {\sum\limits_{l = 1}^{{i_{l} }} {\left[ {\mu_{3l} k_{dl} f_{z} {\text{sin}}\kappa_{r} b_{D} {\Delta }b_{l\alpha }^{0} \omega_{\alpha }^{2} \frac{{\frac{\pi n}{{30}}}}{{\left( {\frac{2\pi n}{{60}}} \right)^{2} - \omega_{\alpha }^{2} }}\left( {\sin \left( {\frac{{4\pi^{2} n}}{{60\omega_{\alpha } }} + \varphi_{1} + \left( {l - 1} \right)\frac{2\pi }{z}} \right)} \right.} \right.} } \\ & \quad \left. {\left. { - \sin \left( {\varphi_{1} + \left( {l - 1} \right)\frac{2\pi }{z}} \right)} \right)} \right] = \frac{{Tf_{z} }}{2\pi }\sum\limits_{ \propto = 1}^{{i_{ \propto } }} {\sum\limits_{l = 1}^{{i_{l} }} {\left[ {\mu_{3l} k_{dl} f_{z} {\text{sin}}\kappa_{r} b_{D} {\Delta }b_{l\alpha }^{0} \frac{{\frac{\pi n}{{30}}f_{\alpha }^{2} }}{{\left( \frac{n}{60} \right)^{2} - f_{\alpha }^{2} }}\left( {\sin \left( {\frac{{2\pi^{2} n}}{{60f_{\alpha } }} + \varphi_{1} + \left( {l - 1} \right)\frac{2\pi }{z}} \right)} \right.} \right.} } \\ & \quad \left. {\left. { - \sin \left( {\varphi_{1} + \left( {l - 1} \right)\frac{2\pi }{z}} \right)} \right)} \right], \\ \end{aligned}$$

wherein: ${\omega }_{\alpha }=2\uppi {f}_{\alpha },$ and ${f}_{\alpha }$ is the frequency [Hz] of vibrations of component no. $\alpha$.

Assuming identical and unchanging values of the coefficients ${k}_{dl}$ and ${\mu }_{3l}$ for all milling cutter teeth, i.e.: ${\mu }_{3l}={\mu }_{3}=const,$ ${k}_{dl}={k}_{d}=const,$ we will obtain a transformed function of the work of cutting forces in the direction of the layer width:

29 $$\overline{L }=\frac{L}{\frac{T{f}_{z}}{2\pi }{\it {\mu}}_{3l} {k}_{d}\mathrm{sin}{\upkappa }_{r}},$$

and then, after taking into account the dependency (28), following cost function:

30 $$\overline{L }=\sum_{\alpha =1}^{{i}_{\alpha }}\sum_{l=1}^{{i}_{l}}{b}_{D}{\Delta b}_{l\alpha }^{0}\frac{\frac{\pi n}{30}{f}_{\alpha }^{2}}{{\left(\frac{n}{60}\right)}^{2}-{f}_{\alpha }^{2}}\left\{\mathrm{sin}\left[\frac{2\pi n }{60{f}_{\alpha }}+{\varphi }_{1}+\left(l-1\right)\frac{2\pi }{z}\right]-\mathrm{sin}\left[{\varphi }_{1}+\left(l-1\right)\frac{2\pi }{z}\right]\right\}.$$

Minimizing the cost function (30), due the natural frequencies ${f}_{\alpha }, \alpha =1, \dots , {i}_{\alpha }$, which results in an estimate of the minimum work of cutting forces in the direction of the width of the layer, makes it possible to predict the best configuration of the workpiece mounting. With the exception of the hard to determine and therefore generally estimated values of ${\Delta b}_{l\alpha }^{0}$, the others are explicitly defined on the basis of the properties of the milling process.

## Results

**Figure 3:**
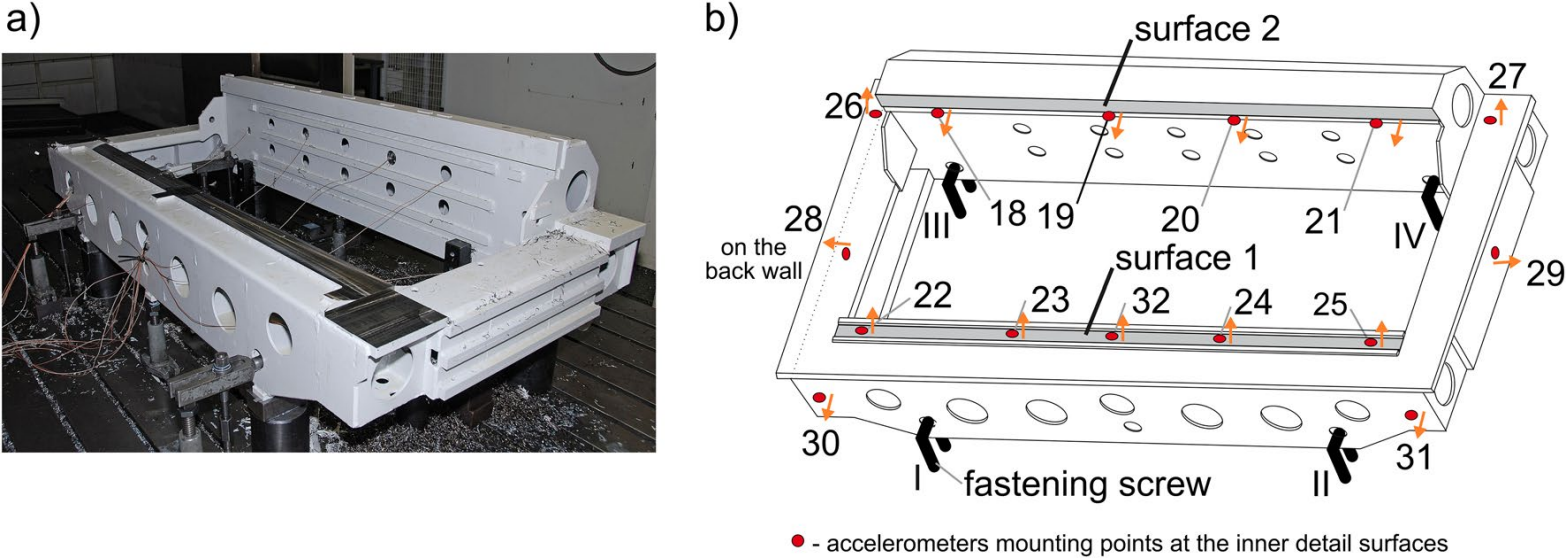
Research object: (**a**) workpiece assembly, (**b**) simplified scheme, accelerometers and fastening screws positions are highlighted.

The experimental research concerned the investigation of the dynamic behavior of a large workpiece (total dimensions 2061 × 1116 × 540 mm, mass 370 kg) made of STW22 03M steel (Fig. 3a). The mechanical properties of such steel according to the European Standard PN–EN 10111:2008 are: yield strength *R*_*p*0.2_ = 170–360 MPa, tensile strength *R*_*m*_ = 440 MPa, elongation A = 22–28 %. And its chemical composition is: C—max. 0.12%, Mn—max. 0.6%, P—max. 0.045%, S—max. 0.045%. The workpiece, selected from the common production program of one cooperating industrial company, was clamped on the table of the MIKROMAT 20 V portal milling center.

**Figure 4:**
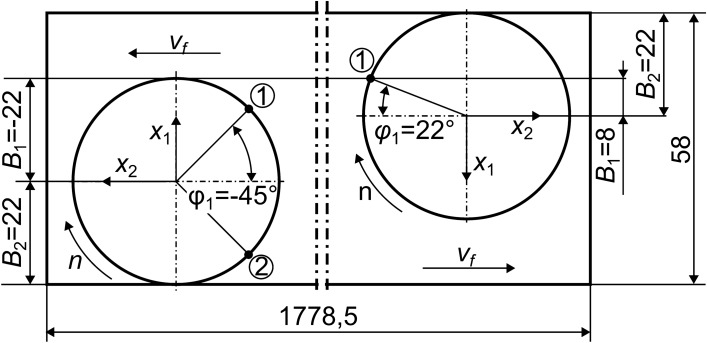
A scheme of face milling of surface 1. The left side of the drawing concerns the full milling pass, and the right side—the down milling pass. The assumed stationary positions of the teeth (CEs) being in contact with the workpiece are marked with numbers in circles.

During face milling of two surfaces 1 and 2 (Fig. 3b), relative vibrations at the conventional point of contact S (Fig. 2) of the workpiece and tool were investigated only for the milled surface 1. The dimensions of the surfaces were: length—1778.5 mm, and width—58 mm (Fig. 4). Full face milling of surface 1 was first performed by the tool starting from the vicinity of accelerometer 22 to accelerometer 25. The next step was down milling by the tool moving in the opposite direction (i.e. starting from the vicinity of accelerometer 25). These two passes formed one complete operation. Milling was performed using a Sandvik R390-044C4-11M060 face milling cutter with a diameter of ϕ44 mm, containing 4 indexable inserts R390-11 T3 08M-PM 1130 with a nose radius of 0.8 mm, a helix angle of the main cutting edge of 12° and a cutting edge angle of *κ*_*r*_ = 90°. In fact, in the construction of the cutting force models (Fig. 2), some simplifications have been made, which, however, did not have a significant impact on the results obtained in the article. And so, the omission of the non-zero nose radius resulted in an error in the radial position of the assumed CE no *l* (i.e. *R*=*D*/2=22 mm) not exceeding 3.6%, and the omission of the non-zero helix angle - an error in determining the maximum immersion angle *φ*_*l*_(*t*) in the direction of the mill axis (along *a*_*p*_=1 mm) not exceeding 1.1°. These simplifications did not affect the estimation of the cutting force work function (30) at all.

In^26^ was described the applied configuration of the measuring equipment and the sampling frequency of all signals was 10 kHz (during cutting experiments) and 15 kHz (during modal tests).

In order to determine the dominant vibration frequencies for different fixing conditions and to select the best one, it is necessary to measure and register the data obtained from modal tests. The measuring points were selected in such a way as to be able to record measuring signals mainly along the milled surface. And so, accelerometers 22, 23, 24, 25 and 32 were placed inside the workpiece along surface 1. Changes between subsequent support configurations consisted in tightening the fastening screws (I–IV) with the same torque measured by dynamometric spanner. The remaining (eleven) supports of the workpiece were not marked in Fig. 3b, and the values of their tightening torques were not changed during experiments.

### Modal identification

Experimental modal tests of the workpiece were performed with the use of modal hammer for a series of impacts induced close to accelerometer 32. The values of FRF were determined using the H3 estimator (the average of standard H1 and H2 estimators). The FRF resolution was 0.5 Hz.

**Figure 5:**
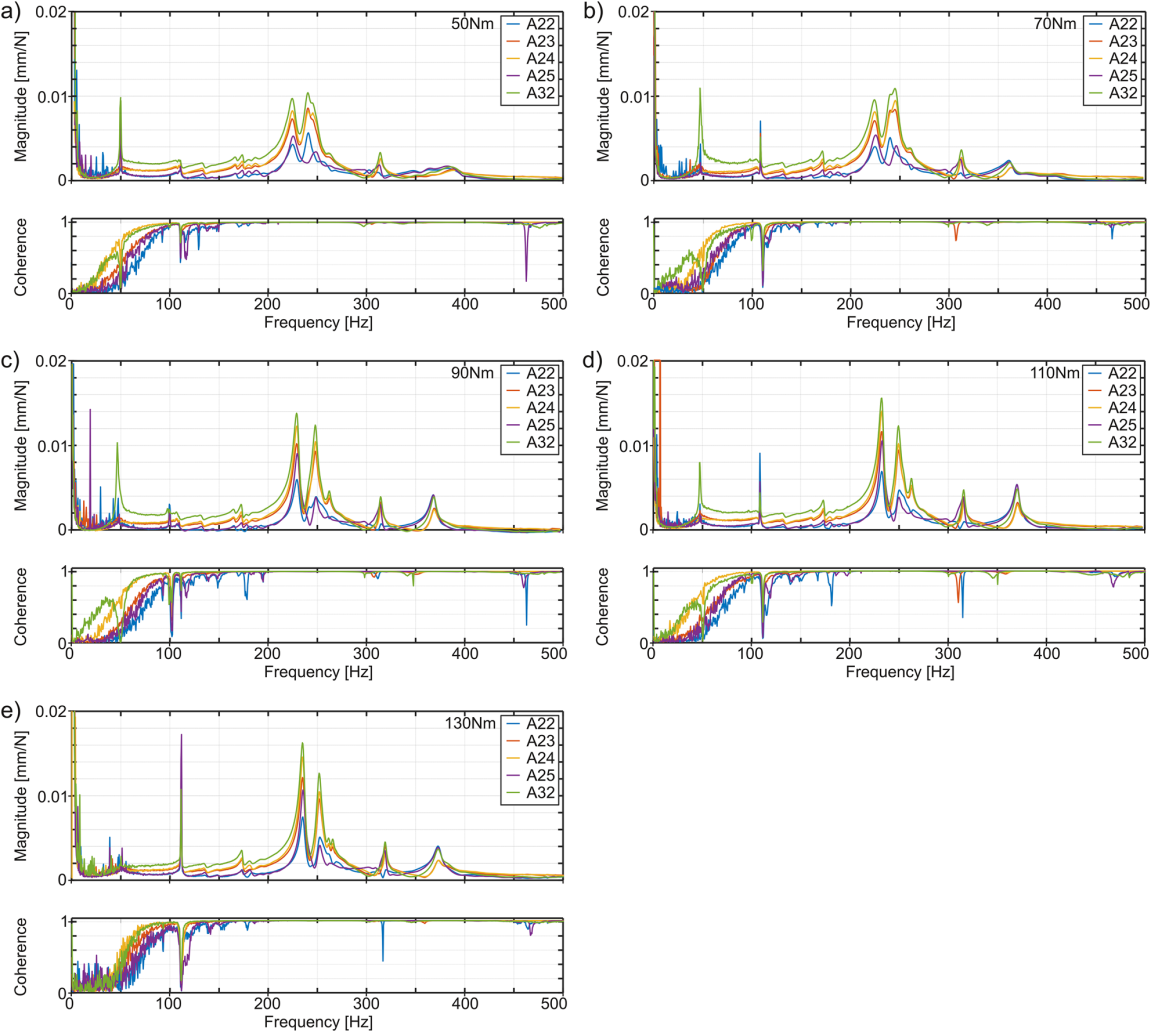
Force-displacements FRFs and coherence functions for surface 1 for all mounting screws tightened with the torque of: (**a**) 50 Nm, (**b**) 70 Nm, (**c**) 90 Nm, (**d**) 110 Nm, (**e**) 130 Nm.

There are shown the force-displacement FRFs and the coherence functions for surface 1 (Fig. 5) when research object was fastened with selected torques ranging from 50 to 130 Nm. The scope of changes in the tightening torques of fasteners of the workpiece, defined in this way, due to the tests carried out under production conditions, resulted from the program of activities of the industrial partner. Hence, the search for the best mounting variant was possible from a finite set of fasteners at various tightening torques used in production practice. Consequently, it was not possible to extend the range of potential tightening torques in the scope of the planned tests. During the analysis of surface 1, the focus was on data from the accelerometer 32 located in the center of the surface. The reason is that the vibrations in the assumed area should be the greatest during machining. Hence, their reduction should be decisive due to the overall level of vibrations when milling the entire surface. The frequencies in the vicinity of the expected harmonic frequencies in relation to the frequency of the teeth entering the material and the other potential natural frequencies of the workpiece were assumed as significant. This selection was made on the basis of knowledge about the planned tool rotation speed *n* = 1300 rpm, the previous modal analysis of the workpiece and the frequency analysis of former machining cases.

From the point of view of the analysis of vibrations occurring during machining, the most important are the forms of low-frequency vibrations, especially those that cause significant displacement values. For the tested workpiece, these frequencies are up to 500 Hz. On the other hand, the coherence function for frequencies below 75 Hz are low and these spectral ranges are practically useless from the point of view of identifying dominant frequencies.

### Minimizing the work of cutting forces

For the purpose of minimizing the work of cutting forces, the related cost function described by the formula (30) was used. In Table 1 are presented, for milling of surface 1, the calculated values of this cost function at the different tightening torques of fastening screws in the range from 50 to 130 Nm, and dominant frequencies in the amplitudes displacements’ spectra, after double integration of the accelerations recorded with accelerometer 32. For an extremely unfavorable configuration of the cutting teeth, here are assumed (Fig. 4), in case of full milling—$z=4$, ${i}_{\alpha }=7$, ${i}_{l}=2$, ${\varphi }_{1}=-45^\circ =-\frac{\pi }{4}$, but in case of down milling—$z=4$, ${i}_{\alpha }=7,$ ${i}_{l}=1$, ${\varphi }_{1}=22^\circ =\frac{22\pi }{180}$. Minimum value of the cutting forces’ work along direction of the cutting layer width was obtained by tightening all four fixing screws with a torque of 50 Nm (bold values) and maximum—by tightening them with a torque of 110 Nm (values underlined).

**Table 1:** Values of the cost function (30) for milling of surface 1.

Tightening torque [Nm]	**50**	70	90	110	130
Spindle speed *n* [rpm]	1300				
*b*_*D*_, ${\Delta b}_{l\alpha }^{0}$ [mm]	1.0, 0.03				
*f*_1_ [Hz]	133.0	133.5	133.5	133.5	134.0
*f*_2_ [Hz]	173.6	174.0	175.0	175.5	172.5
*f*_3_ [Hz]	224.5	226.5	231.5	235.0	234.5
*f*_4_ [Hz]	240.5	248.0	250.5	252.0	251.5
*f*_5_ [Hz]	261.5	263.0	265.0	265.0	265.5
*f*_6_ [Hz]	314.0	315.5	317.0	318.5	318.5
*f*_7_ [Hz]	385.0	365.0	371.0	373.0	372.6
$\overline{L }$[mm^2^ rad/s]	*Full milling*				
	** − 22.99**	− 22.94	− 22.76	− 22.67	− 22.73
$\overline{L }$[mm^2^ rad/s]	*Down milling*				
	− **12.91**	− 12.89	− 12.81	− 12.76	− 12.79

It follows from the above considerations that the best predicted condition for mounting the workpiece on the machine table results from tightening the mounting screws with a torque of 50 Nm.

Some attention should be given to trying to solve the problem by finding the overall contribution of the FRFs at the tooth passing frequency based on the modal testing results, and then finding the minimum value by comparing the FRF values at different clamping torques. However, modal FRF tests are performed on a workpiece that has not yet been machined. Then the FRF maxima correspond only to the natural frequencies of the object, and the amplitude corresponding to the frequency of the cutting edge entering the material and its harmonics may be located in another, less recognizable place of the characteristic. The possibility of its determination depends on: sensitivity of the FRF characteristic and spectrum resolution. The sensitivity of the FRF characteristic determined in this way to changes in the fixing conditions in places that do not correspond to the maxima of this characteristic is low and thus prevents a reliable identification of the best fixing conditions. Especially since the characteristic is reproducible for the same mounting conditions, but only in terms of the frequency values corresponding to the maxima. However, it is not repeatable in terms of FRF amplitudes. In addition, the limited resolution of the spectrum (on the order of 0.5 to 2 Hz, depending on the object and measurement parameters) seriously reduces the chance of “hitting” the FRF characteristic in the amplitude corresponding to the frequency of the cutting edge entering the material. And the rounding to the abscissa corresponding to the nearest one resulting from the spectral resolution may indicate another, non-optimal fixture. Moreover, since the vibrations of the tool–workpiece are supported by the work of the cutting forces, depending on the instantaneous thickness and the instantaneous layer width (formula (30)), all the frequencies occurring in this formula are important, not only those related to the frequency of the cutting edge entering the material and its harmonics. The estimated work of the cutting forces depends on all of these frequencies.

### Implementation of the face milling process for different cases of the workpiece mounting

In order to assess the accuracy of predicting the best conditions for fixing the workpiece, signals of vibration acceleration during the process of face milling of the surface of a large-size workpiece were recorded (Fig. 3b). Then, the displacement plots were obtained by double integration of the recorded signals. The depth of cutting was *a*_*p*_ = 1 mm, the rotational speed of the tool was *n*=1300 rpm, and the feed speed was *v*_*f*_ = 600 mm/min. During the measurements, a very low level of vibrations was observed, apart from the tool entry and exit zone which did not exceed 2 g (acceleration) and 2 µm (displacement).

**Figure 6:**
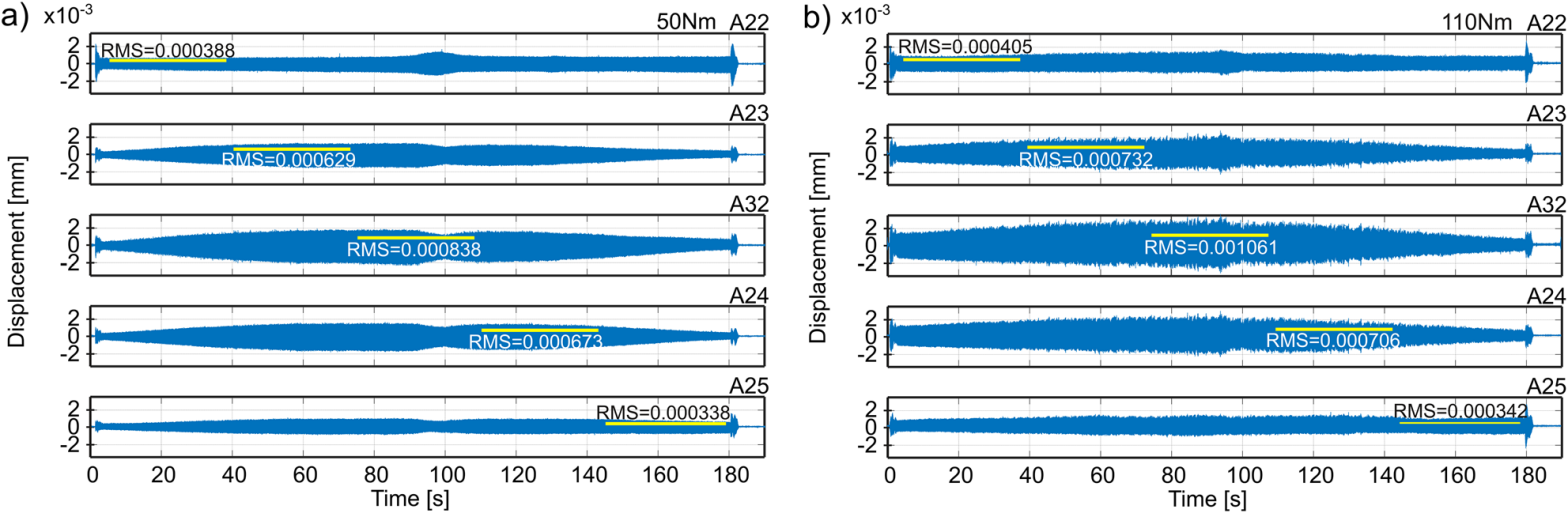
Displacements of vibrations of the workpiece during full milling, obtained on the basis of measurements from accelerometers located along surface 1 at tightening the mounting screws with a torque of: (**a**) 50 Nm, (**b**) 110 Nm.

**Figure 7:**
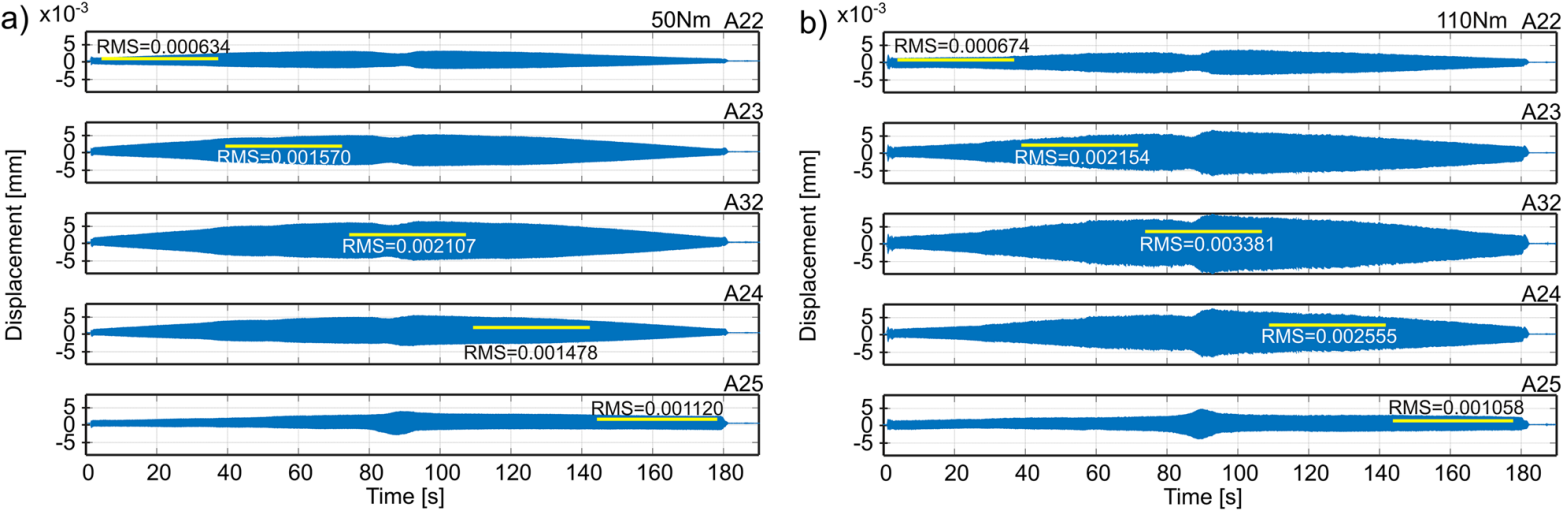
Displacements of vibrations of the workpiece during down milling, obtained on the basis of measurements from accelerometers located along surface 1 at tightening the mounting screws with a torque of: (**a**) 50 Nm, (**b**) 110 Nm.

In Figs. 6 and 7 are shown the time plots of vibration displacements for example variants of fixing workpieces, where Root Mean Square (RMS) values are marked for the areas recognized conventionally for the vicinity of a given accelerometer. The displacements were determined by double integration of the acceleration signal (the signal filtered with an ideal high-pass filter with a cut-off frequency of 25 Hz) for each of the 5 accelerometers placed along the machined surface.

With regard to the application of RMS, one could of course consider using other quantities to evaluate the vibration level in a face milling process. It should be noted, however, that the analyzed process is non-stationary and strongly non-linear, which results in a complex state of relative vibrations of the tool-workpiece. Therefore, other evaluation methods, based on e.g. in steady state frequency analysis, they are not applicable here. The RMS of displacements is the best and most accurate measure of the vibration level assessment in the problem under study. Its value is strongly correlated with the parameters characterizing the quality of the machined surface. The higher the RMS value, the worse the geometric accuracy and surface roughness are^14^.

The RMS results of the vibration displacements are summarized in Table 2. The obtained results of the tool-workpiece vibration measurements in the face milling process confirm the accuracy of predicting the best fixture of the workpiece under the condition of minimizing the work of cutting forces in the direction of the layer width. For full milling of surface 1, the prediction results are consistent with the RMS values of displacement from accelerometer 32 (the best RMS 0.838 µm versus 1.217 µm for the adverse scenario). In the case of down milling, in addition to the readings of accelerometer 32 (the best RMS value 2.107 µm compared to 3.381 µm for the adverse variant), this compliance is additionally confirmed by the average RMS displacements obtained on the basis of the readings of all accelerometers on the surface (the best RMS value 1.382 µm compared to 1,964 µm for the adverse case). The worst results were obtained for the configuration of supports tightened with a torque of 90–110 Nm, and the best - with a torque of 50 Nm.

**Table 2:** RMS values of the vibrations’ displacements during full and down milling of surface 1 for different fixing conditions. Best results are shown in bold, but adverse results are underlined.

Tightening torque [Nm]	Full milling					Down milling				
	50	70	90	110	130	50	70	90	110	130
**Sensor no**	**Displacements [**${\varvec{\mu}}$** m]**					**Displacements [**${\varvec{\mu}}$** m]**				
22	0.388	0.528	0.415	0.405	**0.217**	1.120	1.326	1.036	1.058	**0.620**
23	**0.629**	0.737	0.904	0.732	0.663	**1.478**	2.009	2.121	2.555	1.949
32	**0.838**	1.001	1.217	1.061	0.983	**2.107**	2.774	2.893	3.381	2.624
24	0.673	0.702	0.771	0.706	**0.592**	**1.570**	2.042	2.068	2.154	1.678
25	0.338	0.332	0.288	0.342	**0.194**	0.634	0.876	0.734	0.674	**0.480**
**Average**	0.573	0.660	0.719	0.649	**0.530**	**1.382**	1.805	1.770	1.964	1.470

## Conclusion

An innovative method of solving the problem of vibration suppression during milling of large-size details by predicting the best conditions for clamping the workpiece on the milling machine table is developed in the paper. A successful solution is obtained at the off-line stage, just before the routine milling process, based on a mechanistic model of the cutting process and determination of the dominant amplitude peaks in the spectra obtained as results of modal tests.

Consideration of the stationary positions of the cutting tool with a uniform teeth pitch, in relation to the workpiece in extremely unfavorable cases, turned out to be useful from the point of view of the search for optimal conditions for clamping the workpiece. It was not a significant obstacle to replace the time-varying milling process with a description that ignored the structure configuration changes over time. Despite the adopted assumptions regarding the stationarity of the computational model of the milling process, the minimization of the cost function, which result in the estimation of the minimum work of cutting forces in the layer width direction (30), enables effective prediction of the best configuration of the workpiece mounting.

The obtained results of tool–workpiece vibrations in the face milling process, evaluated on the basis of the RMS values of vibrations in time domain, confirm accuracy of predicting the best conditions for fixing the workpiece. The effectiveness of the tool-workpiece vibration supervision was confirmed in the process of industrial milling of large-size details, based on the clamping conditions determined in the off-line approach.
